# Retraction: Does Development Assistance for Health Really Displace Government Health Spending? Reassessing the Evidence

**DOI:** 10.1371/annotation/4c5252d3-2fd5-4ff2-b3d9-ed197690f877

**Published:** 2012-06-29

**Authors:** Rajaie Batniji, Eran Bendavid

Retraction: Batniji R, Bendavid E (2012) Does Development Assistance for Health Really Displace Government Health Spending? Reassessing the Evidence. PLoS Med 9(5): e1001214. doi:10.1371/journal.pmed.1001214

The authors, Rajaie Batniji and Eran Bendavid, retract their PLoS Medicine essay (1) due to errors in statistical model choice and reporting. Batniji and Bendavid state: “On May 8, 2012, we published an essay that contained a reanalysis of data used by Lu et al. (2) to examine whether development assistance for health leads to displacement of public health spending by recipient governments. In the essay we indicated that we used a fixed effects linear regression, while in Table 4 we reported the results of an analysis using a linear regression without controlling for fixed effects. Upon discovery of this mistake, which resulted from a miscommunication between the authors, we immediately informed PLoS Medicine and moved to correct the record. Our essay concluded that the findings in the analysis by Lu et al. were strongly influenced by outliers and questionable data. However, when our analysis is done using a linear regression with fixed effects, the results show a similar effect size and significance level to that indicated in the original article by Lu et al. As a result of this error, we now believe the following statements in our essay lack justification:

1. “the association between DAH and displacement of government health expenditures is not robust after exclusion of a small subset of data.” 

2. “The trends [in the analysis by Lu et al.] are driven by outliers…” 

3. “While there does appear to be an association, it is too tenuous, too dependent on problematic model selection, and inconsistent.””

The PLoS Medicine editors agree with the need for the Retraction and accept the authors’ explanation of their error. During the editorial handling of the essay, the paper was reviewed twice by our statistician: once following peer review by content experts and revision by the authors, and again following a second author revision and before acceptance. We apologize to readers for any inconvenience caused by the publication of this article in the journal. We have invited short perspectives from Batniji and Bendavid and from Lu et al. to expand upon evidence for aid displacement in health.

References:

1. Batniji R, Bendavid E (2012) Does Development Assistance for Health Really Displace Government Health Spending? Reassessing the Evidence. PLoS Med 9(5): e1001214. doi:10.1371/journal.pmed.1001214

2. Lu C, Schneider MT, Gubbins P, Leach-Kemon K, Jamison D, Murray CJL (2010) Public Financing of Health in Developing Countries: A Cross-National Systematic Analysis. Lancet 375: 1375-1387.

